# Relationship between Emotional Eating, Consumption of Hyperpalatable Energy-Dense Foods, and Indicators of Nutritional Status: A Systematic Review

**DOI:** 10.1155/2022/4243868

**Published:** 2022-05-18

**Authors:** Cristina Elizabeth Fuente González, Jorge Luis Chávez-Servín, Karina de la Torre-Carbot, Dolores Ronquillo González, María de los Ángeles Aguilera Barreiro, Laura Regina Ojeda Navarro

**Affiliations:** Master's Program in Comprehensive Clinical Nutrition, Faculty of Natural Sciences, Campus Juriquilla, Autonomous University of Queretaro, Av. de las Ciencias S/N, Juriquilla, QRO 76320, Mexico

## Abstract

People's health is closely linked to their diet. Diet can be defined as the set of foods that are consumed in a day, and it is susceptible to being altered by various factors, such as physiological, environmental, psychological, and social. These, in turn, can be affected by an inadequate diet and/or a dysregulation of emotions. Emotions are an immediate response by the organism informing it of the degree of favorability of a certain stimulus or situation. Moods are similar to emotions but more intense and prolonged. Some studies indicate that the consumption of hyperpalatable energy-dense foods may be related to emotional eating. Emotional eating is characterized by the excessive consumption of hyperpalatable energy-dense foods, rich in sugars and fats, in response to negative emotions. But several reports also indicate that emotional eating may be associated with the presence of positive emotions, so further analysis of the available information is necessary. Consuming higher amounts of hyperpalatable energy-dense foods can lead to the accumulation of energy in the body that results in an increase in body weight, as well as other associated diseases. Obesity is the world's leading diet-related health problem. The objective of this work was to carry out a systematic review of the available literature using the Cochrane methodology, in accordance with the PRISMA guidelines, to evaluate the relationship between emotional eating, the consumption of hyperpalatable energy-dense foods, and indicators of nutritional status. An exhaustive search in different databases yielded 9431 scientific articles, 45 of which met the inclusion criteria. This review underscores the fact that knowing and understanding the reasons why people consume hyperpalatable energy-dense foods and the possible connection with their emotional eating can provide key data for improving and personalizing patients' nutritional treatment. This in turn can encourage compliance with treatment plans to improve people's health and quality of life using an interdisciplinary approach.

## 1. Introduction

The formation of eating behavior, defined as the attitudes and psychosocial factors related to the selection and ingestion of food, develops during the first years of life. Children learn what, when, and how much to eat by appropriating beliefs and attitudes transmitted to them and from their cultural and home environment. Parents and/or caregivers play an important role in shaping children's early experiences with food and feeding. All this generates certain emotional experiences that can be related to emotional eating and food consumption patterns in adulthood, which may supersede the physiological reactions related to the hunger-satiety cycle [1–6].

The literature suggests that the expression of emotion is regulated by personality traits in an environment/subject binomial. That is, the context determines the type of emotion expressed. Each individual has different emotional scenarios throughout life which are modified at each stage [7]. In this paper, we refer to emotions as a set of defined experiences, which for the purposes of simplicity, will be reduced to two categories: positive (satisfactory, reflecting positive stimuli) and negative (rejection, reflecting discomfort or disgust). Emotions are complex and multifactorial immediate responses, which translate into physical and psychological changes in the individual, and these in turn can significantly influence thought and behavior. They are an immediate response by the organism informing it of the degree of favorability of a stimulus or situation. Moods, on the other hand, are considered reminiscent of emotions but are more diffuse states, which appear for no specific reason and are prolonged over time [8]. It is necessary to distinguish between emotional state and a person's inherent character or personality traits. For example, being nervous about the result of a scholarship you applied for is not the same as being a nervous person. In the first case, it is a temporary situation that ends when you get the news of the scholarship. In the second case, it is a personality trait that accompanies you in different situations and throughout your life.

Hunger and satiety are regulated by the hypothalamus, which is the region of the brain located below the thalamus, and the focal point of a complex network of neural circuits involved in the monitoring of the internal environment (e.g., energy metabolism and temperature regulations). Two of its nuclei (the lateral and ventromedial nuclei) participate in the regulation of food intake and functioning, respectively, to control the onset and termination of eating. Damage to the lateral hypothalamus significantly reduces food and water intake and produces anorexia; damage to the ventromedial hypothalamus generates uncontrollable hunger and obesity. Hypothalamic circuitry monitors a variety of hormonal and other neurochemical factors that are released from the digestive tract and adipose tissue and serve to regulate energy consumption [9]. This complex regulation mechanism is made up of two systems: one anabolic and the other catabolic. The first, also called orexigenic, is responsible for regulating the maintenance or increase in body weight by stimulating food intake, triggering mechanisms that induce hunger and appetite and inhibit energy expenditure. The second, anorexigenic, mechanism is responsible for regulating the maintenance or reduction of body weight, stimulating mechanisms that increase energy expenditure, and decreasing food intake [9]. The hypothalamus, together with the corticolimbic system and the dorsoventral nuclear complex, controls the homeostatic and nonhomeostatic regulation of appetite. It is also a critical component of the hypothalamic-pituitary-adrenal axis (HPA) with projections upon neurons producing central molecular signals that determine eating behavior. This network is also related to the expression of emotions and disorders that may occur in response to stress. Activation of the HPA stimulates the synthesis and release of cortisol, which at normal levels contributes to the normal regulation of many important biological functions but becomes harmful when chronically elevated [10]. Some studies [11–13] point out that dopamine and serotonin participate in the regulation of appetite (defined as the internal motor that drives the search, choice, and ingestion of food and which it is regulated through the relationship between homeostatic and hedonic mechanisms), in dietary balance and in food reward; a low level of these chemicals is related to emotional eating. Dopamine has also been linked with addictive behavior.

Homeostatic control of food intake is strongly influenced by hedonism, the reward system, and eating experiences [9]. In relation to food intake, diet is defined as the set of all foods that an individual habitually consumes. For evaluation purposes, it is analyzed for 24-hour periods, that is, every day. A diet must be complete, sufficient, varied, balanced, and safe in order to allow for the proper growth and development of an individual [14]. A diet that meets these criteria ensures an adequate nutritional status and therefore a good state of health. An evaluation of nutritional status includes an assessment of anthropometric, biochemical, clinical, and dietary indicators. For example, common anthropometric indicators are BMI, abdominal circumference, waist circumference, hip circumference, waist-to-height ratio, and others that are commonly used to characterize the nutritional status of an individual or population. The BMI value—the result of dividing weight (kg) over height (m) squared—has been used to stratify a population or individual as low weight, normal weight, overweight, or obese. As the assessment of all indicators (anthropometric, biochemical, clinical, and dietary) in research studies is complicated in terms of time, analysis, and cost, researchers usually use only one of these to characterize the nutritional status of a population, recognizing the limitation that this poses. Similarly, dietary indicators, such as food energy intake versus individual energy needs, allow for three possible scenarios: a negative energy balance, a neutral energy balance, and a positive energy balance. The latter indicates that the amount of energy an individual obtains from the food they consume exceeds their energy needs, and therefore, that excess energy accumulates in the body in the form of triacylglycerides. For this reason, analysis of diet is essential in assessing the nutritional status.

It is known that diet is susceptible to being altered by various physiological, environmental, psychological, cultural, and social factors and consequently that it involves the emotions and moods. Many foods are known for provoking a reaction of hedonism—that is, they generate a stimulus (wanting or liking a food) food—in response to which the individual may be unable to stop eating, or want to continue eating, even without feeling homeostatic hunger, since these foods generate pleasure. Satiety participates at the physiological level, but hedonic hunger sidesteps this response, as the availability and palatability of food in the environment have an important effect on whether the food will be desired and consumed [15]. Palatability refers to the pleasure experienced when eating a specific food. This sensation is determined by the organoleptic characteristics of the food, that is, its taste, smell, color, and texture, and this in turn will determine choice and intake. Fats are known to be responsible for the flavor, texture, and aroma of foods, and are also related to overeating. Overeating can result in a positive energy balance that, when sustained over a certain period of time, translates into an increase in body weight. Sugar is another powerful component of palatability, encouraging its consumption. The combination of these two appealing components is what makes food “hyperpalatable” [9]. To encourage preference and consumption of certain products, the food industry injects excessive amounts of fats, sugars, and sodium in its recipes to make hyperpalatable energy-dense foods [16].

Various authors define emotional eating as the direct consequence of negative emotions, that is, eating in response to negative emotions instead of physical hunger [17–22]. Emotional eating can be the result of a confusion between internal states of hunger and satiety, and physiological symptoms related to emotions [23]. The response is related to two theories: the first is the set-point theory and the second is the positive incentive theory. The first theory explains natural physiological demands and has nothing to do with emotions or satiety. The second theory is related to eating behaviors in which foods are reinforcers (positive/negative stimuli). The positive incentive theory incorporates culture, sociodemographic environments, and socialization that respond to the evolutionary history of the humankind [7, 24]. This would explain why emotional experiences can stand in for physiological reactions in the hunger-satiety cycle.

Food impacts the response and expression of positive and negative emotions, and in turn, emotions can have a powerful effect on food choices [25]. Also, some studies report that positive emotions are one of the sociocultural factors that influence eating behavior [5, 26]. The most frequent positive emotions in people who are not emotional eaters are joy, relief, love, enjoyment, happiness, and pleasure. This type of person may consume hyperpalatable energy-dense foods only for pleasure. In counterpart, when a person consumes hyperpalatable energy-dense foods high in fat and sugars in response to negative emotions, they often feel hedonic pleasure and instant reward [27]. The negative emotions that have been studied the most in relation to eating behavior are anxiety, sadness, loneliness, worry, boredom, anger, stress, depression, and anger [28, 29]. Emotional eating has been reported to be positively correlated with body mass index (BMI) and can result in weight gain [30–32]. Increase in body weight is a risk factor for the development of diseases that compromise nutritional status and health in general. Obesity is the world's leading diet-related health problem. In the literature, only one review article was found in 2019 [33] analyzing the association between emotions and eating behavior in normal and overweight adult populations. Further research is necessary into the relationship between the consumption of hyperpalatable foods and emotions, and their impact on the health of individuals, taking into account any age or sex differences. The objective of this work was to carry out a review of the literature to better understand the relationship between emotional eating, the consumption of hyperpalatable energy-dense foods, and indicators of nutritional status.

## 2. Method

This review was carried out in accordance with the PRISMA guidelines. Articles were searched in major electronic databases such as PubMed, Science-Direct, EBSCO, Redalyc, and Dialnet. The search strategy was limited to articles indexed in English and Spanish from 2013 to 2021 using combinations of the following keywords: emotional eating, negative emotions, and food intake; emotions and food; stress and food intake; emotional eating and mood.

Inclusion criteria were defined according to PICOS (i.e., population, intervention, comparator, results, and study design). Randomized controlled trial, cross-sectional, case-control, and longitudinal full-text articles were considered for this review. Studies that mentioned variables related to nutritional status (BMI, waist circumference) and emotional eating were also included. Only articles from journals indexed in JCR, Scopus, and Scimago were incorporated in this review. Articles were excluded if they were duplicates; if they did not have a complete text; if they focused more on eating disorders; or were published in the form of cases, letters, or comments to the editor. The main selection of articles based on the inclusion criteria was carried out by review of the title and the abstract, followed by a selection of the full text of those articles whose reading of the abstract suggested potential eligibility.

**Figure 1:**
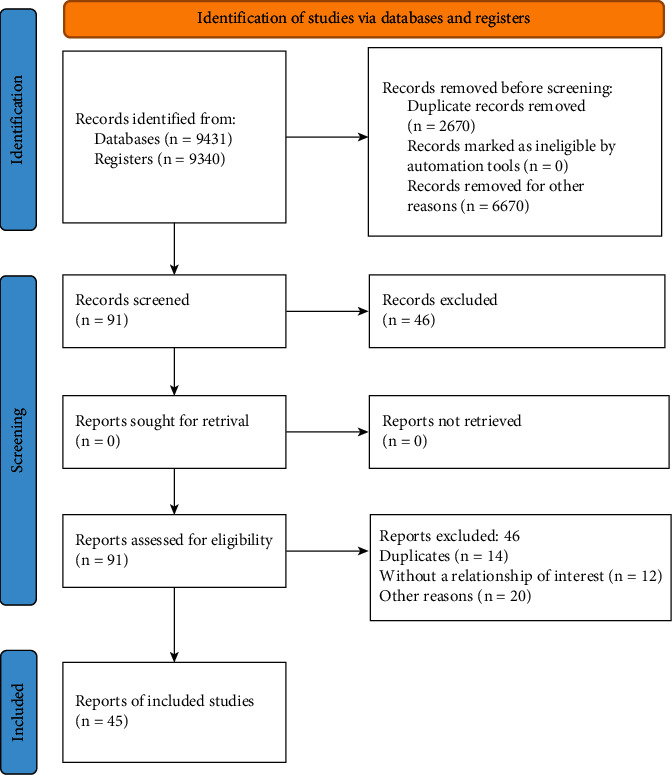
PRISMA 2020 flow diagram.

A total of 9431 studies were identified from the electronic databases. 9340 articles were excluded after reading the title and abstract. Of the 80 articles selected by title and abstract, 46 were excluded for different reasons, mainly: studies that dealt with eating disorders, duplicate studies (14), articles that did not report a relationship of interest (12), and for other reasons (20) as articles without any measure of emotions. Thus, a total of 45 articles remained for the review (Figure 1). According to Scimago Journal & Country Rank platform, 26 articles are from Q1 journals, 10 articles from Q2, and 9 articles from Q3.

## 3. Relationship between Negative Emotions and the Consumption of Hyperpalatable Energy-Dense Foods

Today, in various parts of the world, hyperpalatable energy-dense foods (rich in sugars and fats), generally of industrial origin, have become increasingly available and affordable. Over time, the habit of consuming them has taken hold, displacing the consumption of healthier foods such as fruits and vegetables [34].

Eating behavior is driven not only by physiological processes such as hunger and satiety but also by hedonic eating or hunger, which is the motivation created by exposure and repeated consumption of very tasty energy-rich foods. Many times, food is eaten for the pleasure it produces, or for its taste, but also seeking the feeling of satisfaction or pleasure when consuming it. In this way, a negative emotional state prior to eating the food can be masked or made more tolerable. Frank [13] conducted a randomized controlled trial with 34 28-year-old women in order to investigate the influence of dopamine depletion on food reward processing. He concluded that altered dopamine reward pathways could be one of the several contributors to overeating and obesity. The increased availability and affordability of hyperpalatable energy-dense foods, combined with the pleasure or satisfaction that these can provide reinforces an obesogenic environment, which predisposes the person-to-weight gain and associated diseases that can coexist with deficiencies of certain nutrients.

Several studies carried out in adults indicate a relationship between emotional eating and the consumption of hyperpalatable energy-dense foods (Table 1). Bennett et al. [35] studied perceptions of 19-year-old college students (8 women and 8 men) about their emotional eating behaviors. They reported that stress and boredom were related to emotional eating and hyperpalatable energy-dense foods. Likewise, they reported that during episodes of emotional eating, they would choose what they defined as unhealthy foods. The authors mentioned that learning to manage emotions during this stage of transition from university to adult life is critical for the development of healthy and sustainable eating behaviors.

Emotion-driven eating has been associated with stressful events in life. Raspopow et al. [36] conducted a study among 103 undergraduate women to examine the association between food intake and changes caused by stressors. They found that emotional eaters were more likely to eat in response to an acute stressor when that stress was coupled with subjective feelings of hunger. Stress was described by Reichenberg et al. [37] as a state in which environmental demands exceed an individual's resources, including their coping skills, provoking reactions at the cognitive-emotional, behavioral, or physiological levels. Stress is considered a negative emotion, as are anxiety, anger, and boredom [38, 39], and is frequently associated with emotional eating.

Camilleri et al. [19] conducted a study in 7378 men and 22,862 women to examine the association between emotional eating and the consumption of high-energy snacks, evaluating the two-way interaction of emotional eating with gender, and depressive symptoms. They reported that this association is more pronounced in women with depressive symptoms than in those without, although this association was found in men without depressive symptoms. In another study, Crockett et al. [39] carried out a study in 552 adults aged 18 to 46 years to determine whether eating in response to negative emotions was a generalized phenomenon, and whether boredom influenced eating behavior. The researchers found a general association between the disposition to negative emotions and eating in response to a variety of emotions.

Other studies carried out in adult women also reported a relationship between emotional eating and the consumption of hyperpalatable energy-dense foods. Bongers et al. [40] conducted a randomized trial in 42 women aged 19–27 years to examine whether emotional eaters overeat simply in response to negative emotional cues, or to other cues as well. The authors mentioned that negative emotions are associated with higher intake in response to other signals. Van Strien et al. [41] ran three studies in women to determine whether the desire to eat in response to positive emotions is an “obese” eating style, that is, a style more prevalent in people with obesity. The first study (188 women) evaluated the moderating effect of subjective well-being in the association of BMI with the desire to eat in response to negative emotions (DEBQ-E). The second study (832 women) evaluated whether the desire to eat in response to positive emotions had the same correlation with body mass as the desire to eat in response to negative emotions. The third study evaluated 203 women and an overweight subsample (*n* = 40) to determine whether self-reported desire to eat in response to positive emotions predicted actual food intake, and whether it did more so than their self-reported desire to eat in response to negative emotions. The authors concluded that only the desire to eat in response to negative emotions constitutes an “obese” eating style.

Braden et al. [38] conducted a study in 189 adults to examine whether specific types of emotional eating (eating in response to depression, anxiety/anger, boredom, and positive emotions) were associated with a variety of psychological variables (global psychological well-being, symptoms of eating disorders, and emotional regulation) and physical health. They reported that depression, anxiety/anger, and boredom were more frequently associated with poorer psychological well-being, more symptoms of eating disorder, and greater difficulties with emotional regulation. Positive emotions were not related to the outcome variables. The authors concluded that there are unique patterns of correlation between specific types of emotional eating and psychological outcomes.

Aguiar-Bloemer et al. [42] investigated the influence of emotions evoked by life events on food choices in 48 normal weight and overweight women between 25 and 42 years of age. They concluded that common life problems can trigger food consumption in the presence of high availability. Likewise, they reported that food consumption increased in both groups after experiencing negative emotions.

In a study carried out in 306 Spanish adults, López-Galán et al. [43] evaluated whether emotional eating had a positive or negative influence on consumer preferences for certain food products. In this study, it was concluded that the emotional eating style negatively impacts shopping behavior, which supports the hypothesis that negative emotions trigger excessive consumption of hyperpalatable energy-dense foods. In a cross-sectional study of 200 women, López-Cepero et al. [44] examined the association between emotional eating and overeating among Latinos and the possible mediating role of energy-dense food consumption in this relationship. The study concluded that emotional eating was positively associated with overeating and that intake of energy-dense foods partially mediated this association.

In a laboratory study by Schnepper et al. [3] in 69 women, emotional overeating was investigated by exposing people to personalized emotion induction while displaying images of palatable foods. Outcome variables indexed signal reactivity to food images through image ratings, facial expressions, and brain reactivity. The influence of emotional condition (negative, neutral) and individual differences (self-reported tendencies or traits toward emotional or restrained eating) on outcome variables was assessed. The authors investigated the extent to which the trait of emotional or restricted eating and emotional states predict changes in appetitive response to food images. They concluded that those who define themselves as restrained eaters attribute more attentional resources to food in negative emotional states but lack the corresponding appetitive responses.

Similarly, two studies carried out in university students found a positive relationship between negative emotions and the ingestion of hyperpalatable energy-dense foods (Table 2). In a laboratory study by Mantau et al. [45] involving 179 university students, participants' emotions were manipulated (negatively or positively) and their real food choices were observed, to evaluate the possible determinants of emotional eating. The researchers reported that situational factors such as stress and psychological factors are more relevant in explaining food choices in response to a negative affective state. Ashurt et al. [46] conducted a study in 663 first-year college students living in residence halls to examine associations between emotions—either negative (sad, stressed, and tired), positive (happy, energized, and relaxed), or apathetic (bored, meh)—and their food choices (sweets, salty snacks/fried foods, fruits/vegetables, pizza/fast food, sandwiches/wraps, meats/proteins, pasta/rice, and cereals). They concluded that negative and positive emotions were significantly associated with food choices. They also mentioned that first-year college students who experience negative emotions may be less motivated to choose healthy foods and more motivated to consume hyperpalatable energy-dense foods. This is because food is often used as a distraction to divert attention from negative emotions.

There are also some studies in children and adolescents that explored the association between negative emotions and the intake of hyperpalatable energy-dense foods (Table 3). Nguyen et al. [47] conducted a study in 617 high school students in order to identify the students' dietary choices associated with emotional eating. The authors reported an association between emotional eating and the frequency of intake of sweet foods with a high-energy content. This relationship has also been found in more recent studies in children and adolescents, which concur with or reaffirm the Nguyen's findings, showing the same association between emotional eating and greater consumption of energy-dense snacks, and in particular sweet and high-fat foods (hyperpalatable). For example, Tate et al. [21], in their study of 978 fourth-grade girls, examined two facets of stress (self-efficacy and perceived helplessness) and food consumption mediated by emotional eating. In the study, they concluded that perceived helplessness may predict emotion-driven eating and unhealthy snacking. Another study was carried out by Jalo et al. [1] in a sample of 5426 children to evaluate the associations between self-reported emotional eating, health behaviors (dietary patterns, physical activity, duration of sleep, and TV watching) with the BMI. In this study, a positive association was reported between emotional eating and unhealthy diet patterns. These authors also hypothesized that emotional eating is most likely a learned behavior.

## 4. Relationship between Positive Emotions and the Consumption of Hyperpalatable Energy-Dense Foods

Most of the research has focused on the relationship between negative emotions and eating behavior. However, some studies also report a relationship between positive emotions and the consumption of hyperpalatable energy-dense foods (Table 4). Desmet et al. [48] examined the emotions experienced by healthy adults in response to tasting or ingesting food. The study mentioned that although all emotions may arise from time to time in response to eating or tasting food, pleasant emotions were reported more frequently than unpleasant ones. That is, satisfaction, enjoyment, and desire were experienced more frequently, while sadness, anger, and jealousy were less frequent. The authors proposed five different sources of food emotions to represent the various eliciting conditions reported: sensory attributes, experienced consequences, anticipated consequences, personal, or cultural meanings, and the actions of associated agents.

Evers et al. [49] conducted a study in 68 university adults to investigate the role of positive emotions as a trigger for food intake. The researchers found that positive emotions evoke higher caloric intake. Peña-Fernández et al. [5] conducted a study in 819 university students to identify the emotions experienced by a non-clinical sample while eating. The authors concluded that pleasant emotions are one of the sociocultural factors that motivate eating behavior. It is known that in most cultures, food is present in important celebrations such as birthdays, weddings, religious ceremonies, and other celebrations. These are generally considered happy events, generating positive emotions, so this makes meals more enjoyable and eating more hedonic than homeostatic. In this way, the diet, when affected by emotion, leads to a greater consumption of food.

Reichenberg et al. [37] conducted a study in 59 women to determine what effect stress, negative, and positive emotions had on two key facets of eating behavior—eating based on taste and eating based on hunger—in daily life. These researchers found that higher stress led to decreased taste-eating, which is in line with physiological stress models. Time pressure during eating resulted in less taste—and more hunger—eating. Their findings emphasized the importance of individual differences in understanding eating behavior in daily life, and also suggest that sometimes the choice of hyperpalatable energy-dense foods can also be due to a lack of time and planning to buy and prepare healthier foods.

Donofry et al. [26] conducted a randomized trial in 96 women to examine whether negative emotions reinforce the association between dietary restraint and attention to food bias. They found that positive emotions may be associated with better signal processing of palatable foods. In a study involving children aged 9 to 10 years and in young adults aged 19 years, Moss et al. [50] explored the relationship between positive and negative emotions and eating behavior in this population, together with the effects of eating styles, whether emotional or external. They reported that children felt the urge to snack more in response to positive emotions, while young adults did so in response to negative emotions. This suggests that emotion-driven eating behavior may change with age and the environment in which people develop.

## 5. Relationship between Hyperpalatable Energy-Dense Food Intake, Emotional Eating, and Indicators of Nutritional Status

Emotional eating is a determinant of excessive consumption of hyperpalatable energy-dense foods, which can lead to an increase in body weight and BMI, as mentioned in various studies (Table 5). One of these was Pontes et al. [51], who studied the emotional behavior of 99 adults, specifically the relationship between their food intake and their emotions, with the aim of creating personalized dietary guidelines based on healthy eating habits. In the same vein, another study [52] surveyed a sample of 227 overweight and obese college students to examine associations between decreased emotional eating and weight loss success. They also assessed whether participation in a behavioral weight loss intervention program was associated with a greater reduction in emotional eating over time. The authors noted that decreased emotional eating was associated with weight loss (in terms of BMI). Lazarevich et al. [30] conducted a study of 1453 college students to examine the association between symptoms of depression, emotional eating, and BMI. They also evaluated emotional eating as a mediator between depressive symptoms and BMI. The study reported that helping people to properly manage emotions while detecting depression in vulnerable individuals are crucial for reducing the risk of obesity. Bénard et al. [53] studied a sample of 9974 men and 39,797 women from the NutriNet-Santé cohort study to analyze the moderating influence of “consideration of future consequences” and impulsivity on the relationship between emotional eating and BMI. The authors reported that impulsivity and “consideration of future consequences” moderated the association between emotional eating and body weight. This study emphasized the importance of taking psychological traits into account in obesity prevention. Konttinen et al. [54] conducted a study in 3735 adults aged 25 to 74 years to analyze whether emotional eating mediated associations between symptoms of depression and change in BMI and abdominal circumference over 7 years. The authors reported that eating in response to negative emotions mediated positive associations between depression and increased BMI and abdominal circumference over 7 years.

Also of concern is the fact that excess intake of hyperpalatable energy-dense foods may result in the development of other diseases such as diabetes, heart disease, dyslipidemia, and others, causing an unhealthy nutritional state and affecting quality of life. But other factors also influence dysregulated eating behavior, which has led some researchers to turn their attention to issues of lifestyle, which include physical activity and the duration of sleep. In a study of 138 children and adolescents, Shriver et al. [55] examined associations between childhood emotional regulation, adolescent weight status, negative body image, and emotional eating. These researchers found that the regulation of childhood emotions plays a critical role in shaping subsequent emotional eating, and that dysregulated eating behavior is closely associated with increased adiposity and increased risk of obesity in adolescence and adulthood. The same occurs with stress (negative emotion), which together with emotional dysregulation has a direct impact on eating behavior and an indirect impact on BMI. This conclusion was mentioned by Czepczor et al. [56] who evaluated the impact of behaviors related to eating and emotional functioning on BMI in 298 adults.

On the other hand, it is known that dopamine is one of the key agents for food reward and the control of food intake, which is why Frank et al. [13] mentioned in their study that altered dopaminergic reward pathways could contribute to overeating and therefore to the development of diseases such as obesity.

Better understanding the relationship between emotions, emotional state, personality traits, and food intake, can help nutritionists, physicians, and psychologists work together to develop comprehensive strategies for developing personalized dietary treatments to reduce body weight and mitigate the rate of abandonment [51].

## 6. Discussion and Conclusion

**Figure 2:**
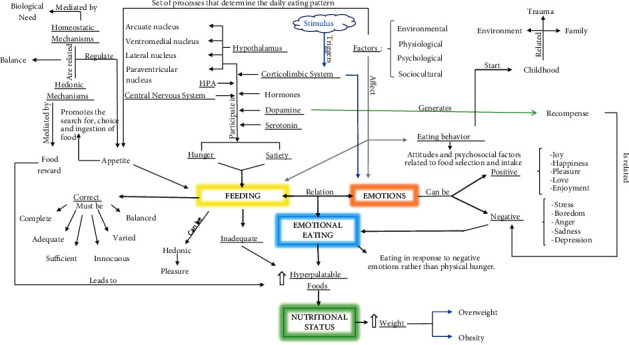
Main actors involved in food, hunger-satiety cycle, emotions, and nutritional status.

As observed in the studies included in this review, emotional eating has been related to unhealthy dietary patterns in which subjects consume an excess of hyperpalatable energy-dense foods rich in fat and sugar. This pattern has been observed in both men and women at different stages of life. Both positive and negative emotions play an important role in the choice, purchase, and consumption of food. Negative emotions have the greatest influence on eating behavior, which is formed from childhood and continues into adult life. Determining the relative weight of emotions (both positive and negative) versus the role of hunger-satiety cycle regulated at the hypothalamic level, in the consumption of food, is a vast and complex matter. An approximation of how these factors intervene in eating, emotions, and nutritional status is provided in Figure 2.

Bilici et al. [57] studied a sample of 2434 adults to determine the relationship between emotional eating behavior, the tendency to eat palatable foods, and various risk factors, concluding that negative emotions are highly influential in eating behavior. When people experience negative emotions, they are motivated to choose unhealthy hyperpalatable energy-dense foods. Stress, boredom, and depression were the emotions most frequently identified as relating to higher food intake. Furthermore, it has been established that depression is more prevalent in women than in men. Note that depression is an emotional health disorder and is not considered an emotion *per se*, but for practical reasons, it has been grouped into negative emotions. As Van Strien et al. mentioned, the desire to eat in response to negative emotions can be considered an “obese” eating style [41]. In contrast, positive emotions are associated with better signal processing of palatable foods. These emotions represent a sociocultural factor in eating behavior, favoring excessive hedonically motivated food consumption, in which people may be unaware of the real reason for the increase in their consumption of hyperpalatable energy-dense foods [5].

One limitation of this review was the wide variety of methodologies and instruments used by the authors to assess emotions, eating behavior, and food consumption. Also, several authors used self-reported emotional eating indicators, which may not accurately reflect people's actual eating behavior under natural conditions. Most of the studies that were found to be related to nutritional status use BMI and waist circumference as indicators. More recent studies, however, have found that the waist–hip ratio is more sensitive than these. Research is necessary into multidisciplinary treatments for weight control that report on strategies for managing emotions, taking into account the individual's personality traits.

Human beings manifest what they have learned in a specific context and culture. The literature we reviewed suggest that emotions and food are both culturally influenced, and in a constant process of socialization. From a nutritional point of view, emotional eating fosters a positive energy balance derived from excessive intake of hyperpalatable energy-dense foods. This causes the accumulation of energy in the form of triacylglycerides in adipose tissue. When this situation is repeated daily, it triggers a series of metabolic alterations that lead to overweight, obesity, and comorbidities such as hypertriglyceridemia, hypercholesterolemia, cardiovascular diseases, diabetes mellitus, kidney diseases, and even deficiencies of certain nutrients. Eating behavior may be altered by a person's emotional state, as they respond to external situations in their day-to-day existence. Eating behavior may be a part of daily life but it may also be used as a means of dealing with negative emotions or stress, rather than a reaction to physical hunger. In an imbalanced diet, nutritional requirements are not adequately covered; a healthy diet is complete, varied, balanced, safe, sufficient, and adequate. Knowing and understanding the reasons for the consumption of hyperpalatable energy-dense foods and their connection with the person's emotions can provide key data for use in personalizing and improving compliance with treatment. This should be carried out in a multidisciplinary way, with a focus on managing emotions and stress, as well as on changing dietary behavior, in order to improve the patient's health and quality of life.

## Figures and Tables

**Table 1:** Studies that report a relationship between negative emotions and hyperpalatable energy-dense food intake in adults.

Author	Location	Population	Study design	Findings
Bennett et al. [35]	Rhode Island	19-year-old college students	Prospective cohort study	Positive relationship
Raspopow et al. [36]	Canada	Undergraduate women	Analytical cross-sectional study	Positive relationship
Camilleri et al. [19]	France	Adults	Prospective cohort study	Positive relationship
Crockett et al. [39]	North Dakota	Men and women	Descriptive cross-sectional study	Positive relationship
Bongers et al. [40]	Maastricht, The Netherlands	Women	Randomized controlled trial	Positive relationship
Van-Strien et al. [41]	Germany	Women	Comparative cross-sectional study	Positive relationship
Litwin et al. [6]	Philadelphia	Women	Randomized controlled trial	Positive relationship
Braden et al. [38]	Ohio	Adults	Randomized controlled trial	Positive relationship
Aguiar-Bloemer et al. [42]	Brazil	Women	Experimental study	Positive relationship
López-Galán et al. [43]	Spain	Adults	Experimental study	Positive relationship
Lopez-Cepero [44]	Massachusetts	Women	Descriptive cross-sectional study	Positive relationship
Schnepper et al. [3]	Austria	Women	Experimental laboratory study	Positive relationship

**Table 2:** Studies that report a relationship between negative emotions and hyperpalatable energy-dense food intake in food choices in adults.

Author	Location	Population	Study design	Findings
Mantau et al. [45]	Central Europe	University students	Randomized experimental study	Positive relationship
Ashurst et al. [46]	Arizona	University students	Comparative cross-sectional study	Positive relationship

**Table 3:** Studies that report a relationship between negative emotions and hyperpalatable energy-dense food intake in children and adolescents.

Author	Location	Population	Study design	Findings
Nguyen-Michel et al. [47]	Los Angeles, USA	Secondary students	Descriptive cross-sectional study	Positive relationship
Tate et al. [21]	Southern California	4th grade girls and Hispanics	Randomized experimental study	Positive relationship
Jalo et al. [1]	12 countries and 5 continents	4th grade girls and Hispanics	Comparative cross-sectional study	Positive relationship

**Table 4:** Studies that report a relationship between positive emotions and hyperpalatable energy-dense food intake in adults.

Author	Location	Population	Study design	Findings
Desmet [48]	Holland	Adult men and women	Comparative cross-sectional study	Positive relationship
Evers et al. [49]	Netherlands	Men and women	Randomized controlled experimental study	Positive relationship
Peña et al. [5]	Mexico City	22-year-old university students	Comparative cross-sectional study	Positive relationship
Reichenberger et al. [37]	Germany	Adults	Comparative cross-sectional study	Positive relationship
Donofry et al. [26]	Pittsburg	Women	Randomized controlled experimental study	Positive relationship
Moss et. al. [50]	United Kingdom	Adults	Comparative cross-sectional study	Positive relationship

**Table 5:** Studies that report a relationship between the intake of hyperpalatable energy-dense foods, emotional eating, and indicators of nutritional status.

Author	Location	Population	Study design	Findings
Pontes et al. [28]	Madrid, Spain	Adult men and women	Longitudinal study	Determining the relationship between food intake and emotions allows for personalization of the dietary strategy to reduce body weight and lower the quit rate.

Braden et al. [52]	New York	University	Randomized controlled trial	Although decreased emotional eating was associated with greater odds of weight loss success, the gold standard behavioral weight loss treatment for overweight adults did not produce major improvements in emotional eating compared to usual care.

Lazarevich et al. [30]	Mexico City	University	Comparative cross-sectional study	Emotional eating was a mediator between depression and BMI, adjusted for age in both sexes. This finding suggests that the management of emotions should be taken into account in obesity prevention and treatment strategies applied to young adults.

Bénard et al. [53]	France	Adults	Prospective cohort study	Impulsivity and consideration of future consequences moderated the association between emotional eating and body weight status.

Konttinen et al. [54]	Finland	Adults	Prospective cohort study	Eating in response to negative emotions mediated positive associations between depression and increased BMI and WC for 7 years, supporting the hypothesis that emotional eating is a behavioral mechanism linking depression and the development of obesity and abdominal obesity.

Shriver et al. [55]	North Carolina	Children and adolescents	Prospective longitudinal study	The regulation of childhood emotions plays a critical role in shaping subsequent emotional eating into dysregulated eating behavior that has been closely associated with increased adiposity and an increased risk of obesity in adolescence and adulthood.

Czepczor-Bernat, et al. [56]	Poland (Silesia)	Adults	Observational study	Significant relationships were found between (almost all) behaviors related to eating, emotional functioning, and body mass index in adults.

## Data Availability

The data used to support the findings of this study are included within the article.
